# Outbreaks of an Emerging Viral Disease Covary With Differences in the Composition of the Skin Microbiome of a Wild United Kingdom Amphibian

**DOI:** 10.3389/fmicb.2019.01245

**Published:** 2019-06-21

**Authors:** Lewis J. Campbell, Trenton W. J. Garner, Kevin Hopkins, Amber G. F. Griffiths, Xavier A. Harrison

**Affiliations:** ^1^Environment and Sustainability Institute, University of Exeter, Penryn, United Kingdom; ^2^Institute of Zoology, Zoological Society of London, London, United Kingdom; ^3^FoAM Kernow, Penryn, United Kingdom; ^4^College of Life and Environmental Sciences, University of Exeter, Exeter, United Kingdom

**Keywords:** disease, amphibians, microbiome, commensal bacteria, ranavirus, *Rana temporaria*, host-microbe interactions

## Abstract

There is growing appreciation of the important role of commensal microbes in ensuring the normal function and health of their hosts, including determining how hosts respond to pathogens. A range of infectious diseases are threatening amphibians worldwide, and evidence is accumulating that the host-associated bacteria that comprise the microbiome may be key in mediating interactions between amphibian hosts and infectious pathogens. We used 16S rRNA amplicon sequencing to quantify the skin microbial community structure of over 200 individual wild adult European common frogs (*Rana temporaria*), from ten populations with contrasting history of the lethal disease ranavirosis, caused by emerging viral pathogens belonging to the genus *Ranavirus*. All populations had similar species richness irrespective of disease history, but populations that have experienced historical outbreaks of ranavirosis have a distinct skin microbiome structure (beta diversity) when compared to sites where no outbreaks of the disease have occurred. At the individual level, neither age, body length, nor sex of the frog could predict the structure of the skin microbiota. Our data potentially support the hypothesis that variation among individuals in skin microbiome structure drive differences in susceptibility to infection and lethal outbreaks of disease. More generally, our results suggest that population-level processes are more important for driving differences in microbiome structure than variation among individuals within populations in key life history traits such as age and body size.

## Introduction

Emerging infectious diseases represent a major threat to amphibian biodiversity around the globe (Daszak et al., 1999). In recent decades both fungal and viral pathogens have been implicated in population declines and extinctions of multiple amphibian taxa, including toads, newts, and salamanders (e.g., Longcore et al., 1999; Price et al., 2014; Martel et al., 2014). The loss of amphibian species due to these pathogens is an enormous conservation concern (Mendelson et al., 2006; Gray et al., 2015), and developing strategies to mitigate the lethal consequences of amphibian disease is now a major priority. Recent research has highlighted the crucial role of host-associated microbial communities in determining the susceptibility of amphibians to lethal pathogens (Woodhams et al., 2007; Lam et al., 2010; Flechas et al., 2012; Jani and Briggs, 2014; Becker et al., 2015; Kueneman et al., 2016; Rebollar et al., 2016b; Jani et al., 2017), and harnessing the host-protective properties of these microbes may be key to modulating host resistance to infection. However, the vast majority of research to date has focussed on the interactions between the amphibian microbiome and the fungal pathogen *Batrachochytrium dendrobatidis* (*Bd)* (e.g., Bletz et al., 2013; Woodhams et al., 2014; Becker et al., 2015; Kueneman et al., 2016; Antwis and Harrison, 2018), and comparative data from other pathogen groups is lacking (Federici et al., 2015; Harrison et al., 2017). Understanding the mechanisms by which the skin microbiome affects host-pathogen interactions, and the generality of those mechanisms across multiple pathogen groups, requires that we measure how skin microbiome covaries with disease severity in the wild across a broad suite of pathogen types.

Ranaviruses are a globally emergent group of viral pathogens which belong to the genus *Ranavirus* (Miller et al., 2011). Ranaviruses are very large, double stranded DNA viruses which are capable of infecting and causing significant morbidity and mortality in all classes of ectothermic vertebrates (Price et al., 2017). Ranaviruses have been known to cause mass-mortality events in infected populations and complete population mortality in some instances (Green et al., 2002; Wheelwright et al., 2014), subsequently ranaviruses are also thought to pose an extinction threat to amphibians (Earl and Gray, 2014; Campbell et al., 2018a) and are believed to be responsible for greater than 80% declines observed in infected United Kingdom populations of the European common frog (*R. temporaria*; Teacher et al., 2010). Clinical ranavirus infection, termed ranavirosis, is commonly fatal and symptoms often exhibited include; severe dermal ulceration, emaciation, and internal hemorrhage (Cunningham et al., 1996; Balseiro et al., 2009; Miller et al., 2011).

Despite the fact that skin is an important organ of infection in ranavirosis (Cunningham et al., 2007), the protective role of amphibian skin against ranaviral infection has received little research attention, especially compared to the body of work which focusses on amphibian skin – *Bd* interactions. It is known that cutaneous peptides produced by amphibians can inactivate viral pathogens, including ranaviruses (Chinchar et al., 2001, 2004; Holthausen et al., 2017), however data on the relationship between ranaviruses and the amphibian skin microbiome are scant. Developing strategies to mitigate the enormous threat that ranaviruses pose to amphibian biodiversity demands that we address this shortfall in our knowledge. Previous work on captive *R. temporaria* identified that animals with a less diverse skin microbiome were more likely to succumb to ranavirus infection in a controlled infection trial (Harrison et al., 2017). Additionally, Campbell et al. (2018b) found evidence of an interaction between ranavirosis and commensal bacterial communities in the wild. Using contaminant bacterial reads filtered from RNA-Seq data sets, it was shown that microbiome species composition and diversity of *R. temporaria* varied in concert with population history of ranavirosis. However, to date no study has performed a targeted examination of the interactions between amphibian skin microbiome and disease outbreaks due to ranavirus in the wild. Importantly, Campbell et al. (2018b) also demonstrated that ranaviruses are potentially more widespread in the United Kingdom than previously assumed, with ranaviral genetic material being detectable at all *R. temporaria* populations sampled, even those with no history of clinical ranavirosis, suggesting that the occurrence or not of ranavirosis within a population is determined by factors other than mere presence or absence of the causative virus.

Here, we use 16S rRNA amplicon sequencing to perform an extensive field survey of the covariation between ranavirosis and the amphibian skin microbiome. Specifically, we compare traits of the skin microbiome of wild adult *R. temporaria* from populations of known ranavirosis outbreak history, and also quantify the effects of variation in host phenotypic data including the sex, size, and age on skin microbiome structure. Given that ranaviruses appear to be present even in healthy United Kingdom *R. temporaria* populations (Campbell et al., 2018b), if the skin microbiome plays a significant role in protection against ranaviral disease, we would expect to observe an association between skin microbiome structure and disease history, whereby populations that have experienced disease outbreaks are more similar to one another than putatively disease-free populations, irrespective of geographical proximity. Therefore, we sought to test the hypotheses that: (i) microbiome structures detected on the skin of frogs originating from ranavirosis-positive populations would be distinct from those of frogs from disease-free habitats and (ii) that bacterial communities would vary based on age, body size, and sex of sampled frogs.

## Materials and Methods

### Sample Collection

**FIGURE 1:**
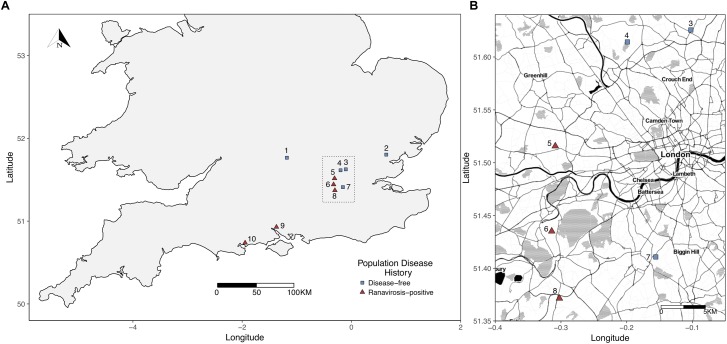
**(A)** Map of sampled populations within the southern United Kingdom. To minimize potential environmental bias, differential abundance analysis, and indicator species analyses were restricted to those populations within the Greater London (area outlined by the dotted black box). **(B)** Closer scale map of populations within Greater London used for differential abundance and indicator species analysis. Populations = 1 – Oxford; 2 – Witham; 3 – Palmer’s Green; 4 – Folkington Corner; 5 – Ealing; 6 – Chessington; 7 – Mitcham; 8 – Tadworth.

Potential ranavirosis-positive populations were drawn from the Frog Mortality Project database of *R. temporaria* populations known to have experienced at least one mass mortality event due to ranavirosis and continued elevated annual mortality (see Price et al., 2016). Our comparative set of disease-free populations were selected from a complimentary database of *R. temporaria* populations that are known to have never experienced disease (see, Teacher et al., 2010 for more detailed selection criteria). All frog populations inhabited urban or semi-urban, permanent ponds located on privately owned land and five geographically interspersed populations of each disease history were selected for inclusion in our study (Figure 1). A subset of the populations used in this study were also studied by Campbell et al. (2018b) who found evidence of ranaviruses even in populations with no history of clinical disease, therefore we used a historical presence or absence of acute ranavirosis to differentiate our disease history groups rather than the detection of ranaviruses themselves.

In the United Kingdom, the onset of *R. temporaria* breeding occurs between January and April annually and is linked to latitude, with more southerly populations breeding earliest. Selected populations were monitored by fieldsite owners throughout the spring breeding season. Each fieldsite was attended on a separate day, when fieldsite owners estimated that the assembled breeding population was at its maximum size (compared to previous annual observations). All populations were sampled between January and March 2015 (see Supplementary Table S1).

As many frogs as possible were captured from each population during a 1-h, mid-morning search window. Frogs were sexed phenotypically by the presence of nuptial pads on the fore limbs of males and the snout to vent length (SVL) of each frog was measured using 0.1 mm scale calipers. Frogs were rinsed by two rounds of immersion in sterile water, then the distal portion of the first digit of a forelimb of each frog was clipped using surgical scissors. To minimize the potential for pain and the possibility of cross infection, a topical disinfectant that contained an analgesic (Bactine; WellSpring Pharmaceutical, FL, United States) was applied to the surgical area before and after the procedure. Surgical equipment was sterilized with 95% ethanol between individuals. Toe clips were placed into separate 1.5 ml micro-centrifuge tubes containing 1 ml of RNA-later nucleic acid stabilization buffer (Ambion, CA, United States) and stored at -80°C. After sampling, all animals were immediately released at the point of capture. The number of individuals sampled per each population varied between 4 (Witham) and 61 (Mitcham and Palmer’s Green) with a mean of 30 animals sampled per site (Supplementary Table S1).

### DNA Extraction

We removed the skin from each toe clip and extracted DNA using a DNeasy Blood and Tissue 96-well DNA extraction kit (QIAGEN, Hilden, Germany) with a modified protocol. To increase the degree to which our extracted DNA represented the true bacterial diversity present on the skin of sampled frogs we added a preliminary digestion step using the enzyme mutanolysin and bead beating. Both of these measures have been shown to increase the efficacy of cell lysis of gram positive bacteria, which are often underrepresented in bacterial diversity assays (Yuan et al., 2012). Skin samples were placed into 1.5 ml micro-centrifuge tubes that contained 177 μl of kit lysis buffer plus 3 μl of 25 KU/ml mutanolysin (Sigma-Aldrich, MO, United States). Five steel lysis beads were added to each tube and the tubes were agitated at 2000 Hz for 4 min using a Qiagen Tissue Lyser (QIAGEN, Hilden, Germany). Skins were then incubated with shaking (180 rpm) at 37°C for 1 h. Following incubation, 20 μl of kit supplied Proteinase K was added to each micro-centrifuge tube and the samples were incubated at 55°C for 5 h. Samples were then transferred into 96-well spin column extraction plates and DNA was extracted according to manufacturer’s instructions.

### Age Determination

The age of each captured frog was calculated using skeletochronology (determination of age by counting growth rings in clipped toe bones) following the protocol of Miaud et al. (1999) with the minor modifications as reported in Campbell et al. (2018a).

### 16S Amplification and Sequencing

Amplicon libraries were generated using a modified version of the dual index protocol published by Kozich et al. (2013). For full details of our library preparation workflow please see our Supplementary Methods File. Subsequently, paired 250 base pair reads were generated on a MiSeq system using a 500 cycle, v2 chemistry, sequencing cartridge (Illumina, CA, United States).

To ensure that we obtained an adequate number of sequencing reads per sample, we sequenced DNA extracts from a maximum of 30 individuals per population. For populations where less than 30 individuals were sampled (*n* = 5), extracts from all frogs were sequenced. For those populations where more than 30 individuals were sampled (*n* = 5), extracts from a random subset of 15 male and 15 female frogs were sequenced. If fewer than 15 females were sampled then all females were sequenced with the remainder of the 30 individuals per site made up of randomly selected male frogs. In total we sequenced the skin bacterial communities of 203 frogs (Supplementary Table S1). Our raw sequence data set contained a total of 21,279,782 individual forward and reverse reads.

### Bioinformatics and Statistical Analyses

We used the R (R Core Team, 2014) package DADA2 (Callahan et al., 2016) to quality screen and trim our amplicon reads. DADA2 was also used to detect sequence variants (SVs) in our read data and to assign genus level taxonomic classification by comparison to the SILVA ribosomal RNA database (Quast et al., 2013). Full details of our quality control and bioinformatic workflow are provided in the Supplementary Methods File. Following quality filtering, and merger of overlapping reads, our final read data set consisted of 9,487,423 consensus reads.

We used the R package phyloseq (McMurdie and Holmes, 2013) to quantify differences in bacterial community structure between sites exhibiting different histories of ranavirosis. We removed one sample originating from Palmer’s Green from the dataset due to complete failure to sequence. Additionally, SVs identified as bacterial species belonging to the genera *Halomonas* and *Pseudoalteromonas* were removed as species from these genera are known contaminants of DNA extraction kits and laboratory reagents (e.g., Lupan et al., 2013; Salter et al., 2014). Any reads assigned as eukaryotic in origin were also removed from our dataset at this stage. To narrow the focus of downstream analysis to the likely most important SVs present in our dataset, we subset our data to contain only those SVs that accounted for greater than 0.1% of our total generated reads (as per Rebollar et al., 2016b). Following the removal of potential contaminants, a total of 28,251 SVs were identified in our read data set. The removal of all SVs that contributed less than 0.1% of our total read set resulted in a final set of the 54 most abundant SVs accounting for a total of 1,160,809 consensus reads. To account for biases introduced by differences in individual library sizes we randomly subsampled (rarefied) all libraries down to the size of the smallest (6,367 reads) using the rarefy function of phyloseq.

### Alpha Diversity

Alpha diversity refers to the taxonomic diversity of bacteria present within a single sample (Jiménez and Sommer, 2017), in this case a single frog. We used phyloseq to calculate the Shannon alpha diversity index of SVs represented by the rarefied library of each sample. We chose to calculate the Shannon diversity index as it is easily converted to an effective number of species, which is a better representation of the true species diversity within a sample and allows for more intuitive interpretation of observed changes in diversity (Jost, 2006). We computed the effective number of species for each sample by calculating the exponent of that sample’s Shannon diversity index (Jost, 2006). To investigate the impact of disease history and other demographic and environmental variables on alpha diversity, we constructed a linear mixed effects regression model (LMER) using the R package lme4 (Bates et al., 2015) with a Gaussian error structure. The effective number of species in each sample was fitted as the response variable with disease history of population of origin (a 2-level factor), as well as the age, SVL, and sex of each individual fitted as fixed explanatory variables. We controlled for variation in effective number of species between populations not explained by any of our fixed effects by the inclusion of population of origin as a random intercept term (Table 2). The significance of each explanatory variable was computed using a stepwise simplification procedure and likelihood ratio test between nested models (Table 2).

### Community Structure (Beta Diversity)

We used the R package vegan (Oksanen et al., 2013) to produce a non-metric multidimensional scaling (NMDS) ordination using Bray-Curtis dissimilarity of the between-sample differences in skin bacterial communities. The vegan package requires that all samples have complete metadata at variables of interest, we therefore removed six samples that we were unable to accurately age and for which all metadata was not available. This resulted in a total of 196 samples used in our analysis of beta diversity (Supplementary Table S1). Ordination was performed across three dimensions (*k* = 3) and yielded a stress of fit value of 0.15. We tested for differences in community structure between sample groups using permutational analysis of variance tests (PERMANOVA) implemented using the adonis function of vegan. We used ordination plots (Supplementary Figures S2–S4) to inform the construction of our PERMANOVA models. Based on divergent group centroids, we fitted population of origin, disease history of population of origin, individual age, and SVL as potential predictors of commensal bacterial community structure. We ran our PERMANOVA for 1,000 permutations.

### Differentially Abundant SVs

To identify individual SVs which were more abundant on the skin of frogs from populations of one disease history type than the other we performed differential abundance analysis using the R package DESeq2 (Love et al., 2014). We were able to use the unrarefied read sets of all samples for this analysis as DESeq2 controls for differences in library size by using a negative binomial mixture model, calculates significant differences in the abundance of SVs using Wald tests, and corrects significance *p* values for multiple testing using the Benjamini-Hochberg correction (McMurdie and Holmes, 2014). This allowed us to detect significant differential abundance between disease history population groups without additional bias that may be introduced by the random subsampling of the rarefaction procedure. However, geographical region of origin has been shown to have a large impact on the species present within the amphibian skin microbiome (e.g., Jani et al., 2017). Therefore, to limit the introduction of bias and possible erroneous detection of differentially abundant SVs due to unrecorded environmental variables beyond our control we performed differential abundance analysis on a geographically restricted subset of our sampled populations. This subset included the three ranavirosis-positive populations and the three disease-free populations that are located within the Greater London area (Figure 1), comprising 138 individuals.

To assess the likelihood of observing similar numbers of differentially abundant SVs due to chance, we performed a permutation analysis. Individual samples were randomly assigned to a disease history group and differential abundance was computed as above. The number of significantly differentially abundant SVs (Benjamini-Hochberg corrected *p* value < 0.05) was calculated and recorded. We repeated this process 1,000 times to obtain a distribution of the number of differentially abundant SVs expected due to chance.

### Indicator Species Analysis

As differential abundance analysis does not account for within group consistency in abundance, we identified the SVs likely to be key drivers of community divergence between disease history groups using indicator species analysis (Dufrene and Legendre, 1997). We calculated the indicator value of each of our 54 most abundant SVs using the indval function of the labdsv R package (Roberts, 2007). An indicator score of 1 would indicate that a species is equally abundant in all samples from one disease history group and effectively absent from samples of the other, whereas an indicator score of 0 would suggest approximately even abundance across samples of both disease history types. We therefore considered SVs with an indicator score of 0.7 or higher to be indicators of disease history groups and those with an indicator score of 0.5–0.7 as detector species (suggestive but not indicative of disease history group; e.g., Van Rensburg et al., 1999; Castro-Luna and Sosa, 2009; Bates et al., 2018). As with differential abundance analysis, indicator species analysis was limited to our geographically refined subset of frog populations located in Greater London.

## Results

### Field Sampling and Individual Biometric Data

We sequenced the skin bacterial communities of 202 individual frogs (Supplementary Table S1), comprising 111 individuals originating from ranavirosis-positive populations (80 male and 31 female frogs), and 91 frogs from disease-free populations (58 male and 33 female frogs). A breakdown of the ages and SVLs for each of these groups are given in Table 1.

**Table 1:** Minimum, mean and maximum age and snout to vent length (SVL) for males and females of each disease history type.

	Age (years)			SVL (mm)		
Sex (Disease history)	Min	Mean	Max	Min	Mean	Max
Male (Ranavirosis-positive	2	4.8	10	47.6	69.5	84.0
Male (Disease-free)	4	6.3	9	69.5	73.3	88.4
Female (Ranavirosis-positive)	2	5.3	9	55.0	75.9	92.1
Female (Disease-free)	4	6.5	10	60.3	75.5	90.5

*Age was calculated by skeletochronology and SVL was measured using 0.1 mm scale calipers.*

### Alpha Diversity

Alpha diversity was similar between populations with different disease histories. The mean effective number of species (based upon our 54 most abundant sequence variants) was 5.34 (range = 1.76–17.40) in ranavirosis-positive populations and 6.77 (range = 2.17–16.36) in disease-free populations. When controlling for variation among populations in our LMER, we found that neither sex, SVL nor disease history explained a significant amount of variation in individual effective number of species score (Table 2).

**Table 2:** Summary of lmer model simplification procedure.

LMER Full model	ENS ∼ history + age + SVL + sex + (1| site)			
Fixed effect	Estimate	Df	Chi^2^	*p*
Age	0.06	6	0.06	0.79
SVL	-0.02	5	0.14	0.71
Sex	0.81	4	2.23	0.12
History	-1.50	3	2.54	0.11

*ENS, effective number of species score; SVL, the body length of each individual in mm; History, disease history of populations, either ranavirosis-positive or disease-free. The reference level is disease-free. Df, degrees of freedom of the model including the parameter being tested.*

### Community Structure (Beta Diversity)

**FIGURE 2:**
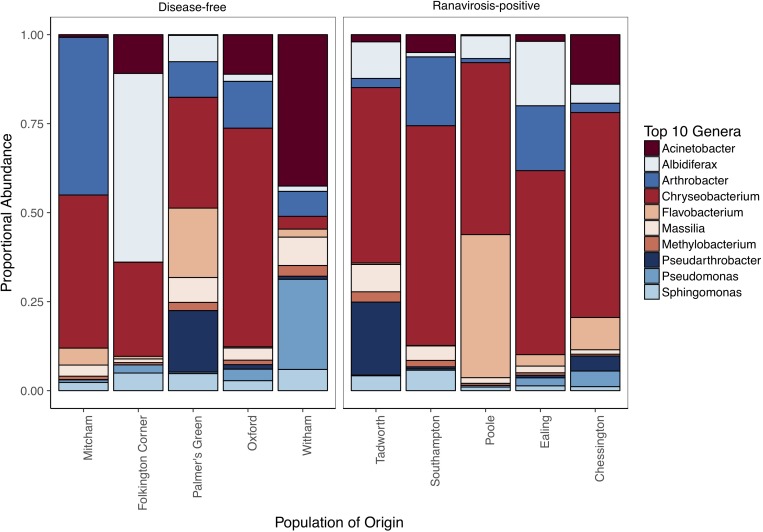
Relative abundance of the top 10 most abundant genera at each of our 10 sampled populations. Populations are grouped by disease history status to allow for visual comparison of abundance patterns.

Of the 1,160,809 consensus reads represented in our 54 most abundant SVs, 644,503 and 516,306 belonged to samples from ranavirosis-positive and disease-free populations respectively. The top ten most abundant genera present in skin commensal communities at both ranavirosis-positive and disease-free populations consisted of the same ten genera, however the rank order of abundance differed between the two disease history groups (Figure 2 and Supplementary Table S5). Relative abundance of individual genera varied between sites (Figure 2 and Supplementary Table S5) but overall, frog skin microbiomes at both ranavirosis-positive and disease-free populations were dominated by SVs belonging to the genus *Chryseobacterium*. Reads classified as *Chryseobacterium* species accounted for 50 and 37% of all reads at ranavirosis-positive and disease-free populations respectively (Figure 2 and Supplementary Table S5).

**FIGURE 3:**
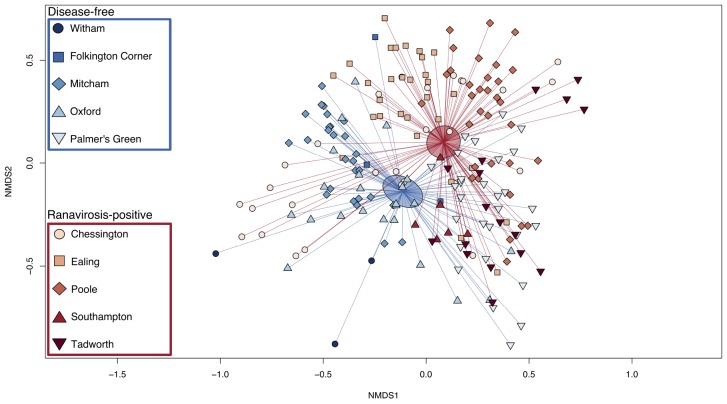
Bray-Curtis NMDS plot of skin microbiome similarity. The microbial community of each individual *R. temporaria* is represented by a point. The shape of the point differentiates individuals from different populations. Ranavirosis-positive populations are shown in red hues and disease-free populations in blue hues. Each point is connected to the averaged point (centroid) of microbial community structure for its respective disease history group. Shaded ellipses represent the 95% confidence interval around the disease history group centroid.

Ordination plots and PERMANOVA results showed that the most important predictor of commensal bacterial community structure was the population from which a frog originated (Figure 3; PERMANOVA; *p* = 0.001; *R*^2^ = 0.37). However, disease history of populations was also a statistically significant predictor of bacterial community structure (Figure 3; PERMANOVA; *p* = 0.001; *R*^2^ = 0.08). Neither individual SVL or age were found to significantly explain variation in skin microbiome structure (PERMANOVA; *p* = 0.60, *R*^2^ = 0.003 and *p* = 0.85, *R*^2^ = 0.002 respectively).

### Differential Abundance and Indicator Analysis

**FIGURE 4:**
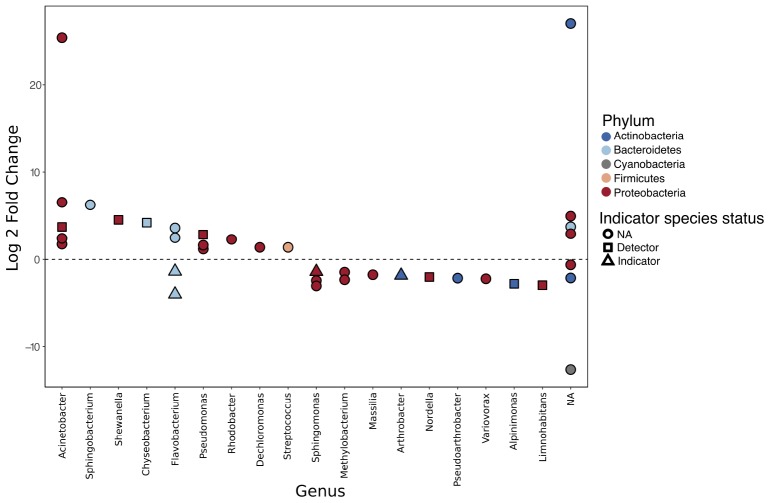
Differentially abundant sequence variants (SVs) identified using DESeq2. A positive log 2 fold change indicates that an SV is enriched in ranavirosis-positive populations and a negative log 2 fold change that the SV is enriched in disease-free populations. The results of our indication species analysis are overlaid. Circular points denote an SV with an indicator species score of < 0.5, suggesting poor association between that SV and a disease history group. Square points denote SVs with an indicator score of 0.5–0.7. Such SVs are considered detector species and have a suggestive but not indicative relationship with the disease history group in which they are enriched. Triangular points indicate SVs with an indicator species score of 0.7 or greater. These SVs are considered indicator species of the disease history group in which they are enriched.

Differential abundance analysis of the 54 most abundant SVs using DESeq2 identified 37 SVs that were significantly differentially abundant between disease history groups following correction for multiple testing. Of these, 20 were found to be enriched in ranavirosis-positive populations and 17 were found to be enriched in disease-free populations, when compared to populations of the opposite disease history status. Differentially abundant SVs were found to belong to a total of five unique phyla and 18 unique genera, not including seven SVs that could not be classified to the genus level (Figure 4).

A randomisation test revealed that the null expectation of the number of differentially abundant SVs when individuals were randomly permuted between disease history classes was 1.16 SVs (range = 0–18 SVs). Our observed statistic of 37 differentially abundant SVs was therefore significantly higher than expected by chance (*p* = 0.001, Supplementary Figure S6), supporting our conclusion that observed differences in abundance represents real differences attributable to disease history groups.

Indicator species analysis revealed that 11 of the 37 differentially abundant SVs detected using DESeq2 possessed abundance profiles that indicate that they may be driving divergence in community structure between the two disease history groups. No SVs that were enriched in ranavirosis-positive populations compared to disease-free populations had an indicator score of greater than 0.7. However four SVs were ascribed indicator scores greater than 0.5 (Figure 4). These SVs belonged to the genera *Acinetobacter*, *Shewanella*, *Chryseobacterium*, and *Pseudomonas*. Four SVs identified as enriched in disease-free populations compared to ranavirosis-positive populations were classified as indicator species (indicator score 0.7 or greater). These SVs belonged to the genera *Flavobacterium* (*n* = 2), *Shingomonas* and *Arthrobacter*. An additional three SVs belonging to the genera *Nordella*, *Alpinimonas*, and *Limnohabitans* had indicator scores higher than 0.5 (Figure 4).

## Discussion

Growing evidence from various systems supports the existence of host protective effects of host-associated microbial communities (Woodhams et al., 2007; Lauer et al., 2008; Honda and Littman, 2012; Legatzki et al., 2014; Kong, 2015). Ranaviruses appear to be widespread in the United Kingdom, but their detection is not always associated with outbreaks of disease (Campbell et al., 2018b). If the amphibian skin microbiome plays a role in increasing resistance to or lessening the impact of ranavirosis, we would expect to observe consistent differences across populations that have and have not experienced significant outbreaks of disease. We therefore hypothesized that frogs originating from populations with a positive disease history of ranavirosis would possess distinct skin microbiomes compared to frogs that originated at populations that have remained disease-free during the time frame of ranaviral emergence in the United Kingdom.

### No Apparent Link Between Ranavirosis and Skin Microbiome Diversity

We found no convincing evidence that a population history of ranavirosis impacts the alpha diversity of commensal bacterial communities on wild *R. temporaria* skin. The evidence from other amphibian-disease systems on how skin microbiome alpha diversity is impacted by disease is mixed, ranging from strong negative effects (Rebollar et al., 2016b; Bates et al., 2018) through to non-significant negative trends (Federici et al., 2015) and no detectable impact (Woodhams et al., 2007; Jani et al., 2017).

However, previous work on captive *R. temporaria* showed that reduced alpha diversity increased the potential for dysbiosis (harmful perturbations of the microbiome) of the skin microbiome and elevated mortality of metamorphic *R. temporaria* subjected to experimental exposure to a ranavirus (Harrison et al., 2017). Interestingly, Harrison et al. (2017) also found that exposure to a ranavirus directly perturbed the host skin microbiome, highlighting the difficulty in detecting the directionality and mechanisms of observed changes in host microbiome using amplicon sequencing methods. Based on Harrison et al. (2017) we could have expected to find evidence of increased microbiome diversity in wild *R. temporaria* populations with no history of ranavirosis. That we did not detect such a pattern is possibly attributable due to key differences between the methodologies of the respective studies.

Most importantly, Harrison et al. (2017) conducted their experiment under common garden conditions, incorporating individual frogs with relatively uniform skin microbiomes at the start of their experiment. Although this approach undoubtedly increases the ability of such experiments to detect subtle shifts in host microbiome between treatment groups, the inherent simplicity is certainly not reflective of natural systems. There is evidence to suggest that many amphibian species, including *R. temporaria*, selectively enrich their microbiome with particular, locally rare, species of bacteria (Loudon et al., 2014; Walke et al., 2014; Kueneman et al., 2016; Bates et al., 2018), potentially in response to external selection pressures such a pathogens (Jani and Briggs, 2014; Walke et al., 2015; Rebollar et al., 2016b; Bates et al., 2018). However, the environment is known to act as a reservoir of microbial diversity from which an individual’s microbiome can be populated (Belden and Harris, 2007; Loudon et al., 2014) and the catalog of bacterial species present within an environment varies based on numerous abiotic factors (Fierer and Jackson, 2006; Lozupone and Knight, 2007; Sylvain et al., 2016; Ji et al., 2017). It is therefore plausible that detectable variation in alpha diversity of commensal microbial communities attributable to disease history is overwhelmed by variation in alpha diversity due to originating from different populations, which are subject to subtly different abiotic factors, and therefore contain different catalogs of microbial species. It is also possible that variation in abiotic factors of an environment between populations have a direct influence on skin microbiome of *R. temporaria*, as has been hypothesized in other species (Kueneman et al., 2014; Krynak et al., 2016; Longo and Zamudio, 2017). The importance of geographical variation as a predictor of microbiome structure is supported by our finding of unique microbiome structures between our sampled populations and the fact that both population of origin and disease-history group were shown to be significant predictors of microbiome structure by our PERMANOVA. Although we have strong evidence for geographical variation in microbiome structure between our sampled populations, the subtle differences between habitats that cause this variation to arise, regardless of mechanism, are unlikely to be a systematic covariate between our disease history population groups.

It should also be noted that the protective effect of amphibian skin microbiomes in response to *Bd* has been attributed to the presence or absence of key bacterial species within the commensal microbial community (e.g., Jani et al., 2017). It is possible that studies which detect changes in alpha diversity in amphibian skin microbiomes in response to a pathogen presence may be detecting differences in the presence or absence of these particular key species.

### Skin Microbiome of *R. temporaria* Covaries With Disease History of Ranavirosis

We found evidence of systematic differences between the skin microbiome composition of wild *R. temporaria* populations with contrasting disease histories of ranavirosis. Although each population had a distinct microbial signature, populations that had experienced historical ranavirosis outbreaks had more similar skin bacterial communities when compared to putatively disease-free populations. Similar findings based upon population disease history alone have been reported by Rebollar et al. (2016b) in the frog *Craugastor fitzingeri* under infection with Bd.

We have identified several SVs that are both differentially abundant between disease history groups and are found at consistently similar abundances within disease history groups that suggests they may be key in driving such observed divergence. Four SVs belonging to the genera *Pseudomonas*, *Acinetobacter*, *Chryseobacterium*, and *Shewanella* were identified as detector species of ranavirosis-positive populations. Additionally, four SVs belonging to the genera *Flavobacterium* (two SVs), *Sphingomonas*, and *Arthrobacter* were identified as indicator species of disease-free populations. The majority of these genera contain species of bacteria which are both potentially protective and potentially pathogenic (Supplementary Tables S7, S8). Three additional SVs enriched on the skin of frogs from disease-free populations were characterized as detector species. These SVs belonged to the genera *Nodella*, *Alpinimonas*, and *Limnohabitans*. These genera are known to occur on the skin of the North American bullfrog (*Lithobates catesbeianus*; Krynak, 1989) and the egg masses of brown trout (*Salmo trutta*; Wilkins et al., 2015), in fresh water (La Scola et al., 2004) and in alpine glaciers (Schumann et al., 2012), respectively, but little else is yet known about their ecology. Given the conflicting evidence regarding protective or pathogenic effect of most of these genera, as well as the fact that the relationship between a single commensal bacterial species and its host can shift from protective to pathogenic and vice versa in an entirely context dependent manner (Daskin and Alford, 2012; King et al., 2016), it is not possible to be conclusive about the nature of relationship between *R. temporaria* and the bacterial species that we have identified. Considering the protective qualities of several of the genera that we found to be enriched in ranavirosis-positive populations it is plausible that the SVs associated with these genera provide a degree of protection against ranavirosis and are therefore under positive selection at ranavirosis-positive populations. However, several alternative explanations also exist and cannot be disregarded at this stage.

It has been hypothesized that the structure of the amphibian microbiome is a function of host genotype (Becker et al., 2015). Infection with a ranavirus has been shown to reduce population genetic diversity and possibly cause the establishment of assortative mating patterns in British *R. temporaria* populations (Teacher et al., 2009a,b). The divergence in skin microbiome structure that we observe between disease history groups may therefore be an emergent property of different population genetic structures between the two population groups. Additionally, ranaviruses have been shown to form co-infections with other pathogens (Olori et al., 2018) so an alternative explanation could be that the presence of ranavirus within a population is allowing opportunistic pathogenic bacteria to colonize or proliferate on the skin of *R. temporaria* through either a compromised host immune response (Pasman, 2012), a disturbed microbiome (Theriot et al., 2014) or a combination of the two. Like any habitat, resources available on amphibian skin are finite; increases in abundance of one bacterial species (by either of the two mechanisms listed above) will therefore be offset by the reduced abundance of other bacterial species, hence we observed comparative enrichment of some SVs in disease-free populations compared to frogs from ranavirosis-positive populations.

Since it has now been shown that how the microbiome is impacted by a pathogen is dependent on the microbiome’s initial state (Jani and Briggs, 2014; Becker et al., 2015; Harrison et al., 2017; Jani et al., 2017; Longo and Zamudio, 2017) it remains unclear whether the apparent divergence between the microbiomes of frogs from ranavirosis-positive populations and those from disease-free populations is a consequence or cause of their disease history. Therefore, another potential explanation of our observed differences in community structure between the two groups would be that possessing a microbial community structure similar to that observed at ranavirosis-positive populations predisposes a population to disease emergence and that disease has no subsequent impact on microbial community structure (Becker et al., 2015; Jani et al., 2017). If further research supported this postulation, the ability to detect amphibian populations that may be at highest risk of disease outbreaks by their microbiome signature would allow for more targeted conservation efforts.

It should also be noted that we did not find any differentially abundant representatives of the genus *Bacillus*, nor was the genus represented in our top 10 genera by abundance at populations of either disease history. This is a notable discrepancy between our results and the results of Campbell et al. (2018b). This is likely explainable due to the fact that the results of Campbell et al. (2018b) were based on sequence data generated from RNA sequencing and so represent the bacterial species which are most transcriptionally active within the microbiome rather than those with the highest relative biomass, as is represented by 16S sequence data. This raises interesting questions as to whether biomass or transcriptional activity is the most important indicator of a species’ importance within the amphibian skin microbiome. To date few studies have attempted to examine the metatranscriptome (transcriptional profile of whole microbial communities) or metabolomes (profile of metabolite production for whole microbial communities) of amphibian skin (Rebollar et al., 2016a), a targeted investigation into how population disease history may influence the transcriptional activity of the amphibian skin microbiome would prove valuable and timely.

Finally, evidence from studies of humans and mice suggest that sex, body size, and age can influence the microbiome (Yatsunenko et al., 2012; Yurkovetskiy et al., 2013; Langille et al., 2014; Haro et al., 2016), however we found no evidence of such influences here. Neither sex, age, or body size had any significant impact on the alpha diversity or community structure of the *R. temporaria* skin microbiome. Previous studies on amphibians have found no evidence of variation in skin microbial community structure between the sexes (Hughey et al., 2017) but have shown that commensal microbial communities vary significantly between developmental stages (Kueneman et al., 2014; Longo et al., 2015; Bates et al., 2018). Our study is the first to examine the composition of amphibian microbiomes using fine scale age data within adults. The physiological changes that occur during metamorphosis and sexual maturation are much larger and occur over greatly reduced time periods compared to the slow process of annual aging and growth so it is not entirely surprising that the microbiome of amphibians is known to shift more detectably during these events. However, as discussed previously, the composition of the amphibian skin microbiome is known to be heavily linked to the bacterial composition of the environment in which they reside (Belden and Harris, 2007; Loudon et al., 2014). Since we found that the most important predictor of the microbiome structure of our sampled animals was population of origin, followed by population disease history, we cannot exclude the possibility that the relative strength of such population level factors in shaping the skin microbiome overwhelm the fine scale variation in microbiome composition due to additional phenotypic traits. This further suggests that population level factors like the emergence of disease play a large role in shaping host microbiomes. A deeper understanding of the circumstances under which such interactions occur, and ultimately the mechanisms by which they occur, are likely to prove valuable in efforts to mitigating an ever-growing number of deadly wildlife diseases.

## Conclusion and Future Directions

Our data provide evidence of an interaction between the lethal viral pathogens belonging to the genus *Ranavirus* and the amphibian skin microbiome in the wild. Though we demonstrate that *R. temporaria* from populations with a positive disease history of ranavirosis possess a microbial community structure distinct to that of frogs from disease-free populations, the underlying mechanisms of this variation and its implications for host-pathogen dynamics remain to be elucidated. Laboratory survival trials that incorporate animals seeded with skin microbial assemblages representative of both disease history groups and temporal sampling of the microbiome before and after infection with a ranavirus would allow for further investigation of the mechanisms underlying the changes that we observed and the potential protective benefits of commensal skin bacteria under acute ranavirosis. Laboratory trials will also allow us to gain insight into whether a history of ranvirosis is the cause for observed changes to the skin microbiome, or whether the structure of the microbiome itself may predispose individuals to infection. This latter relationship warrants further investigation, as the ability to use skin microbiome structure as a diagnostic tool to identify which species, or populations of a particular species, are most susceptible to disease would be an invaluable aid to conservation efforts directed toward amphibians.

## Ethics Statement

This work was approved by the ethics boards of both the University of Exeter and the Zoological Society of London and conducted under the United Kingdom Home Office project license 80/2466. All field sampling was conducted under the personal Home Office license 30/1030 issued to LC.

## Author Contributions

LC, TG, and XH devised the study. LC conducted the field work. LC and KH performed the laboratory work. LC and XH conducted the bioinformatics and statistical analyses, and wrote the manuscript. All the authors revised the manuscript and approved the final submitted version.

## Conflict of Interest Statement

The authors declare that the research was conducted in the absence of any commercial or financial relationships that could be construed as a potential conflict of interest.
