# Supplementary material for: Outbreaks of an Emerging Viral Disease Covary With Differences in the Composition of the Skin Microbiome of a Wild United Kingdom Amphibian
Source: Front Microbiol. 2019 Jun 21;10:1245. doi: 10.3389/fmicb.2019.01245 (PMC6597677; doi:10.3389/fmicb.2019.01245)
Supplement: Supplementary file 1 [file Table_1.DOCX]

Supplementary Material

| **Table S1:** The number of frogs sampled and sequenced per each population (# sequenced). #beta is the number of frogs that were used in the beta diversity/community composition analysis | | | | | |
| --- | --- | --- | --- | --- | --- |
| **Population** | **Status** | **Month of sampling** | **# sampled** | **# sequenced** | **# beta** |
| Ealing | Positive | March | 46 | 30 | 30 |
| Poole | Positive | January | 43 | 30 | 30 |
| Southampton | Positive | February | 7 | 6 | 6 |
| Chessington | Positive | March | 30 | 28 | 28 |
| Tadworth | Positive | February | 21 | 17 | 17 |
| Mitcham | Disease Free | March | 61 | 30 | 27 |
| Folkington Corner | Disease Free | March | 4 | 4 | 3 |
| Palmer’s Green | Disease Free | March | 61 | 29 | 29 |
| Oxford | Disease Free | March | 26 | 23 | 23 |
| Witham | Disease Free | March | 5 | 5 | 3 |

|  |
| --- |
| **Fig S2:** Bray-Curtis NMDS plot of skin microbiome similarity with **SEX** dependant group centroids. The microbial community of each individual *R. temporaria* is represented by a point. Blue points relate to Male frogs and red to female frogs. Sex was not used as a predictor in our PERMANOVA analysis. |

|  |
| --- |
| **Fig S3:** Bray-Curtis NMDS plot of skin microbiome similarity with **SIZE** dependant group centroids. The microbial community of each individual *R. temporaria* is represented by a point. Frogs were grouped based on their size. Blue points represent frogs with a body length of less than 70 mm and red points frogs with a body length of greater than 70 mm. Size was used as a predictor in our PERMANOVA analysis. |

|  |
| --- |
| **Fig S4:** Bray-Curtis NMDS plot of skin microbiome similarity with **AGE** dependant group centroids. The microbial community of each individual *R. temporaria* is represented by a point. Frogs were grouped depending on their age. Blue points represent frogs less than 5 years old and red points relate to frogs that are older than 5 years of age. Age was used as a predictor in our PERMANOVA analysis. |

| **Table S5**: Top 10 most abundant genera in both disease status groups. Count = number of reads assigned to each genus. % = the percentage of the total reads (represented within the most abundant 54 SVs) from each disease history group accounted for by each genus. Total number of reads represented within the 54 most abundant SVs = 644,503 and 516,306 for ranavirosis-positive and disease-free populations respectively. | | | | | | |
| --- | --- | --- | --- | --- | --- | --- |
|  | **Ranavirosis-positive** | | | **Disease-free** | | |
| **Rank** | **Genus** | **Count** | **%** | **Genus** | **Count** | **%** |
| 1 | *Chryseobacterium* | 322230 | 50 | *Chryseobacterium* | 190793 | 37 |
| 2 | *Flavobacterium* | 91443 | 14 | *Arthrobacter* | 95765 | 19 |
| 3 | *Albidiferax* | 60856 | 9 | *Flavobacterium* | 40922 | 8 |
| 4 | *Arthrobacter* | 45133 | 7 | *Pseudarthrobacter* | 31321 | 6 |
| 5 | *Acinetobacter* | 26819 | 4 | *Acinetobacter* | 25199 | 5 |
| 6 | *Pseudarthrobacter* | 25267 | 4 | *Albidiferax* | 23711 | 5 |
| 7 | *Massilia* | 15734 | 2 | *Massilia* | 22378 | 4 |
| 8 | *Pseudomonas* | 11508 | 2 | *Sphingomonas* | 16764 | 3 |
| 9 | *Sphingomonas* | 11115 | 2 | *Pseudomonas* | 11314 | 2 |
| 10 | *Methylobacterium* | 6105 | 1 | *Methylobacterium* | 7481 | 1 |

|  |
| --- |
| **Fig S6:** Histogram of the number of differentially abundant SVs identified by our 1000 trials conducted by randomly assigning samples to different disease history groups. The vertical red bar indicates the number of differentially abundant SVs identified when true status grouping were used (37). |

| **Table S7 –** Summary of literature presenting evidence of potentially protective effects of bacterial species identified as enriched at ranavirosis positive and disease-free R*ana temporaria* populations. IS = Indicator species status, D = Detector species, I = Indicator species. Host species = the species from which the bacterium in question was sequenced or cultured. Pathogen species = the pathogen from which the bacterium in question is protective. TL = Taxonomic level to which the study was able to identify the bacterium in question, S = Species, G = Genus and F = Family. | | | | | | |
| --- | --- | --- | --- | --- | --- | --- |
| **Potentially protective species** | | | | | | |
| **Ranavirosis positive populations** | | | | | | |
| **Bacterial genera** | **IS** | **Study** | **Study type** | **Host Species** | **Pathogen Species** | **TL** |
| ***Pseudomonas*** | D | Becker et al., 2015 | *In vivo* / *In vitro* | *Atelopus zeteki* | *Batrachocytrium dendrobatidis (Bd)* | F |
|  |  | Federici et al., 2015 | *In vivo* | *Rana italica* | *Amphibiocystidium sp.* | G |
|  |  | Rebollar et al., 2016 | *In vivo* | Various tropical frogs | *Bd* | G |
| ***Acinetobacter*** | D | Flechas et al., 2012 | *In vitro* | 3 *Atelopus* species | *Bd* | G |
|  |  | Antwis et al., 2015 | *In vitro* | *Agalychnis moreletii & A. callidryas* | *Bd* | G |
|  |  | Rebollar et al., 2016 | *In vivo* | Various tropical frogs | *Bd* | G |
|  |  | Muletz-Wolz et al., 2017 | *In vitro* | N/A | *Bd* & *B. salamandrivorans* | S |
|  |  | Antwis & Harrison, 2018 | *In vitro* | *Agalychnis moreletii & A. callidryas* | *Bd* | G |
| ***Chryseobacterium*** | D | Park et al., 2014 | *In vitro* | *Anaxyrus boreas boreas* | *Bd* | S |
|  |  | Muletz-Wolz et al., 2017 | *In vitro* | N/A | *Bd* & *B. salamandrivorans* | G |
| **Disease-free populations** | | | | | | |
| ***Flavobacterium*** | I | Lauer et al., 2008 | *In vitro* | *Hemidactylium scutatum* | *Mariannaea elegans* & *Rhizomucor variabilis* | S |
|  |  | Lam et al., 2010 | *In vivo* | *Rana mucosa, R. sierrae* | *Bd* | S |
|  |  | Federici et al., 2015 | *In vivo* | *Rana italica* | *Amphibiocystidium sp.* | G |
| ***Arthrobacter*** | I | Harris et al., 2006 | *In vitro* | *Plethodon cinereus* & *Hemidactylium scutatum* | *Bd* | G |
|  |  | Park et al., 2014 | *In vitro* | *Anaxyrus boreas boreas* | *Bd* | S |
|  |  | Lam et al., 2010 | *In vivo* | *Rana mucosa, R. sierrae* | *Bd* | S |

| **Table S8 –** Summary of literature presenting evidence of potentially pathogenic effects of bacterial species identified as enriched at ranavirosis positive and disease-free R*ana temporaria* populations. IS = Indicator species status, D = Detector species, I = Indicator species. Host species = the species from which the bacterium in question was sequenced or cultured. Pathogen species = the pathogenic bacterium identified by the study. TL = Taxonomic level to which the study was able to identify the bacterium in question, S = Species, G = Genus | | | | | | |
| --- | --- | --- | --- | --- | --- | --- |
| **Potentially pathogenic species** | | | | | | |
| **Ranavirosis positive populations** | | | | | | |
| **Bacterial genera** | **IS** | **Study** | **Study type** | **Host Species** | **Pathogen Species** | **TL** |
| ***Pseudomonas*** | D | Glorioso et al., 1974 | In vivo | *Rana catesbeiana* | *Pseudomonas sp.* | G |
|  |  | Driscoll et al., 2007 | Review | *Homo sapiens* | *Pseudomonas aeruginosa* | S |
| ***Acinetobacter*** | D | Koren & Rosenberg, 2008 | *In vivo* | *Oculina patagonica* | *Acinetobacter sp.* | G |
|  |  | Federici et al., 2015 | *In vivo* | *Rana italica* | *Amphibiocystidium sp.* | G |
| ***Chryseobacterium*** | D | Bloch et al., 1997 | *In vivo* | *Homo sapiens* | *Chryseobacterium meningosepticum* | S |
|  |  | Pokrywka et al., 1993 | *In vivo* | *Homo sapiens* | *Chryseobacterium meningosepticum* | S |
|  |  | Green et al., 1999 | *In vivo* | *Xenopus laevis* | *Chryseobacterium meningosepticum* | S |
| ***Shewanella*** | D | Pagani et al., 2003 | *In vivo* | *Homo sapiens* | *Shewanella putrefaciens* | S |
|  |  | Paździor, 2016 | Review | Fresh water fish sp. | *Shewanella putrefaciens* | S |
| **Disease-free populations** | | | | | | |
| ***Flavobacterium*** | I | Bernardet et al., 1996 | Review | Fish sp. | *Flavobacterium branchiophilum, F. columnare & F. psychrophilum* | S |
| ***Sphingomonas*** | I | Lin et al., 2010 | *In vivo* | *Homo sapiens* | *Sphingomonas paucimobilis* | S |
|  |  | Richardson et al., 1998 | *In vivo* | Various corals | *Sphingomonas sp.* | G |

**Cited literature.**

Antwis, R. E., & Harrison, X. A. (2018). Probiotic consortia are not uniformly effective against different amphibian chytrid pathogen isolates. *Molecular Ecology*, *27*(2), 577–589. https://doi.org/10.1111/mec.14456

Antwis, R. E., Preziosi, R. F., Harrison, X. A., & Garner, T. W. J. (2015). Amphibian symbiotic bacteria do not show a universal ability to inhibit growth of the global panzootic lineage of Batrachochytrium dendrobatidis. *Applied and Environmental Microbiology*. https://doi.org/10.1128/AEM.00010-15

Becker, M. H., Walke, J. B., Cikanek, S., Savage, A. E., Mattheus, N., Santiago, C. N., … Gratwicke, B. (2015). Composition of symbiotic bacteria predicts survival in Panamanian golden frogs infected with a lethal fungus. *Proceedings of the Royal Society B*, *282*(1805), 20142881. https://doi.org/10.1098/rspb.2014.2881

Bernardet, J.-F., Segers, P., Vancanneyt, M., Berthe, F., Kersters, K., & Vandamme, P. (1996). Cutting a Gordian Knot: emended classification and description of the genus Flavobacterium, emended description of the family Flavobacteriaceae, and proposal of Flavobacterium hydatis nom. nov. (Basonym, Cytophaga aquatilis Strohl and Tait 1978). *Int. J. Syst. Bacteriol.*, *46*(1), 128–148. https://doi.org/10.1099/00207713-46-1-128

Bloch, K. C., Nadarajah, R., & Jacobs, R. (1997). Chryseobacterium meningosepticum:An Emerging Pathogen Among Immunocompromised Adults Report of 6 Cases and Literature Review. *Medicine*, *76*(1), 30–41.

Driscoll, J. A., Brody, S. L., & Kollef, M. H. (2007). The epidemiology, pathogenesis and treatment of Pseudomonas aeruginosa infections. *Drugs*, *67*(3), 351–368. https://doi.org/10.2165/00003495-200767030-00003

Federici, E., Rossi, R., Fidati, L., Paracucchi, R., Scargetta, S., Montalbani, E., … Di Rosa, I. (2015). Characterization of the Skin Microbiota in Italian Stream Frogs (*Rana italica*) Infected and Uninfected by a Cutaneous Parasitic Disease. *Microbes and Environments*, *30*(3), 262–269. https://doi.org/10.1264/jsme2.ME15041

Flechas, S. V., Sarmiento, C., Cárdenas, M. E., Medina, E. M., Restrepo, S., & Amézquita, A. (2012). Surviving chytridiomycosis: Differential anti-Batrachochytrium dendrobatidis activity in bacterial isolates from three lowland species of Atelopus. *PLoS ONE*, *7*(9). https://doi.org/10.1371/journal.pone.0044832

Glorioso, J. C., Amborski, R. L., Amborski, G. F., & Cully, D. D. (1974). Microbiological studies on septicemic bullfrogs (Rana catesbeiana). *American Journal of Veterinary Research*, *35*(9), 1241–1245.

Green, S. L., Bouley, D. M., Tolwani, R. J., Waggie, K. S., Lifland, B. D., Otto, G. M., & Ferrell, J. E. (1999). Identification and management of an outbreak of Flavobacterium meningosepticum infection in a colony of South African clawed frogs (Xenopus laevis). *Journal of the American Veterinary Medical Association*, *214*(12), 1833—8, 1792—3. Retrieved from http://europepmc.org/abstract/MED/10382028

Harris, R. N., James, T. Y., Lauer, A., Simon, M. A., & Patel, A. (2006). Amphibian pathogen Batrachochytrium dendrobatidis is inhibited by the cutaneous bacteria of amphibian species. *EcoHealth*, *3*(1), 53–56. https://doi.org/10.1007/s10393-005-0009-1

Koren, O., & Rosenberg, E. (2008). Bacteria associated with the bleached and cave coral Oculina patagonica. *Microbial Ecology*, *55*(3), 523–529. https://doi.org/10.1007/s00248-007-9297-z

Lam, B. A., Walke, J. B., Vredenburg, V. T., & Harris, R. N. (2010). Proportion of individuals with anti-Batrachochytrium dendrobatidis skin bacteria is associated with population persistence in the frog Rana muscosa. *Biological Conservation*, *143*(2), 529–531. https://doi.org/10.1016/j.biocon.2009.11.015

Lauer, A., Simon, M. A., Banning, J. L., Lam, B. A., & Harris, R. N. (2008). Diversity of cutaneous bacteria with antifungal activity isolated from female four-toed salamanders. *The ISME Journal*, *2*(2), 145–157. https://doi.org/10.1038/ismej.2007.110

Lin, J.-N., Lai, C.-H., Chen, Y.-H., Lin, H.-L., Huang, C.-K., Chen, W.-F., … Lin, H.-H. (2010). Sphingomonas paucimobilis Bacteremia in Humans: 16 Case Reports and a Literature Review. *Journal of Microbiology, Immunology and Infection*, *43*(1), 35–42. https://doi.org/10.1016/S1684-1182(10)60005-9

Muletz-Wolz, C. R., Almario, J. G., Barnett, S. E., DiRenzo, G. V., Martel, A., Pasmans, F., … Lips, K. R. (2017). Inhibition of Fungal Pathogens across Genotypes and Temperatures by Amphibian Skin Bacteria. *Frontiers in Microbiology*, *8*(August), 1–10. https://doi.org/10.3389/fmicb.2017.01551

Pagani, L., Lang, A., Vedovelli, C., Moling, O., Rimenti, G., Pristera, R., & Mian, P. (2003). Soft Tissue Infection and Bacteremia Caused by Shewanella putrefaciens. *Clinical Journal of Microbiology*, *41*(5), 2240–2241. https://doi.org/10.1128/JCM.41.5.2240

Park, S. T., Collingwood, A. M., St-hilaire, S., & Sheridan, P. P. (2014). Inhibition of Batrachochytrium dendrobatidis Caused by Bacteria Isolated from the Skin of Boreal Toads, Anaxyrus (Bufo) boreas boreas, from Grand Teton National Park, Wyoming, USA. *Microbiology Insights*, *7*, 1–8. https://doi.org/10.4137/MBI.S13639.RECEIVED

Paździor, E. (2016). Shewanella putrefaciens - A new opportunistic pathogen of freshwater fish. *Journal of Veterinary Research (Poland)*, *60*(4), 429–434. https://doi.org/10.1515/jvetres-2016-0064

Pokrywka, M., Vlazanko, K., Medvick, J., Knabe, S., McCool, S., Pasculle, A. W., & Dowling, J. N. (1993). A Flavobacterium meningosepticum outbreak among intensive care patients. *American Journal of Infection Control*, *21*(3), 139–145. https://doi.org/10.1016/0196-6553(93)90005-O

Rebollar, E. A., Hughey, M. C., Medina, D., Harris, R. N., Ibáñez, R., & Belden, L. K. (2016). Skin bacterial diversity of Panamanian frogs is associated with host susceptibility and presence of Batrachochytrium dendrobatidis. *The ISME Journal*, *10*(7), 1682–1695. https://doi.org/10.1038/ismej.2015.234

Richardson, L. L., Goldberg, W. M., Kuta, K. G., Aronson, R. B., Smith, G. W., Ritchie, K. B., … Miller, S. L. (1998). Florida’s mystery coral-killer identified. *Nature*, *392*(6676), 557–558. Retrieved from http://dx.doi.org/10.1038/33302
