# Supplementary material for: Outbreaks of an Emerging Viral Disease Covary With Differences in the Composition of the Skin Microbiome of a Wild United Kingdom Amphibian
Source: Front Microbiol. 2019 Jun 21;10:1245. doi: 10.3389/fmicb.2019.01245 (PMC6597677; doi:10.3389/fmicb.2019.01245)
Supplement: Supplementary file 2 [file Table_2.docx]

Supplementary Methods

16S amplification and sequencing

Amplicon libraries were generated using a modified version of the dual indexed amplification method developed by Kozich *et al.* (2013) which amplifies the V4 region of the bacterial 16S rRNA gene. Each sample was assigned a different combination of two indexed primers and PCR amplification was performed in triplicate for each sample. Replicate amplicon libraries were combined and PCR reaction artefacts were removed using the AMPure magnetic bead clean up system (Agencourt Biosciences, Texas, USA).

One microlitre of each cleaned library was pooled into a single sample. The size, and product specificity of the pooled library was analysed on a Tapestation (Agilent Technologies, California, USA). The concentration of three replicated samples of the library pool were quantified on a Qubit fluorometer (Invitrogen, California, USA). Following NaOH denaturation, we prepared a 3.5pm dilution of our library pool and to 950µl of this dilution we added 50µl of an equal molarity of the PhiX sequencing standard (Illumina, California, USA). This sample and standard mix was incubated for two minutes at 96°C followed by five minutes in an ice water bath. We quantified the concentration of each sample within our pooled library by sequencing on a MiSeq machine using the v2 300 cycle Nano chemistry (Illumina, California, USA). MiSeq Nano cartridges produce approximately 1/12^th^ of the sequence data produced by a full MiSeq sequencing run. From this data, we were able to compute the relative abundance of reads per sample. This enabled us to calculate the exact quantity of each sample required to produce a final equimolar pool so that each sample was approximately evenly represented in our final sequencing dataset. From our equimolar pool, paired 250 base pair reads were generated on a MiSeq machine using a 500 cycle v2 reagent kit (Illumina, California, USA).

Read quality control

We used the R (R Core Team 2014) package DADA2 (Callahan et al., 2016) to process our raw sequence data. Reads were quality scored and trimmed at the first appearance of a base with a quality score of two or lower. Forward reads are expected to be of higher quality so forward reads of trimmed length shorter than 240 bases and reverse reads of trimmed length 160 bases or fewer were excluded from our dataset. Reads that contained any non-assigned bases (N) were also excluded, as were reads with an expected error rate higher than two. Any reads that matched the PhiX sequencing standard genome were also removed at the read quality screening stage.

Detection of sequence variants

DADA2 partitions sequencing error in each read from true genetic diversity and categorises reads together into “sequence variants” (SVs), rather than the more commonly used operational taxonomic units (OTUs), which have recently been shown to return unacceptable levels of falsely identified bacterial diversity (Edgar, 2017). The SVs present in our sequence dataset were computed and paired reads were merged into single consensus reads. Chimeric sequences were removed from the dataset. We assigned our SVs to taxonomic groupings at the genus level by comparison with the SILVA ribosomal RNA database (Quast et al., 2013) from within the DADA2 package.

Cited Literature

Callahan, B. J., McMurdie, P. J., Rosen, M. J., Han, A. W., Johnson, A. J. A., and Holmes, S. P. (2016). DADA2: High-resolution sample inference from Illumina amplicon data. *Nat. Methods* 13, 581–583. doi:10.1038/nmeth.3869.

Edgar, R. C. (2017). Accuracy of microbial community diversity estimated by closed- and open-reference OTUs. *PeerJ* 5, e3889. doi:10.7717/peerj.3889.

Kozich, J. J., Westcott, S. L., Baxter, N. T., Highlander, S. K., and Schloss, P. D. (2013). Development of a dual-index sequencing strategy and curation pipeline for analyzing amplicon sequence data on the miseq illumina sequencing platform. *Appl. Environ. Microbiol.* 79, 5112–5120. doi:10.1128/AEM.01043-13.

Quast, C., Pruesse, E., Yilmaz, P., Gerken, J., Schweer, T., Yarza, P., et al. (2013). The SILVA ribosomal RNA gene database project: Improved data processing and web-based tools. *Nucleic Acids Res.* 41, 590–596. doi:10.1093/nar/gks1219.

Team, R. (2014). R: A language and environment for statistical computing. R Foundation for Statistical Computing, Vienna, Austria. 2013.
